# Effects of strength training on conditioned pain modulation in patients with fibromyalgia: a prospective experimental study

**DOI:** 10.1590/1806-9282.20250109

**Published:** 2025-08-08

**Authors:** Letícia Menegalli-Santos, André Pontes-Silva, Mariana Arias Avila

**Affiliations:** 1 Universidade Federal de São Carlos, Department of Physical Therapy – São Carlos (SP), Brazil.

**Keywords:** Pain, Musculoskeletal system, Quality of life, Exercise, Public health

## Abstract

**OBJECTIVE:**

The aim of this study was to evaluate the conditioned pain modulation of patients with fibromyalgia and compare it to that of healthy individuals before and after 3 months of strength training.

**METHODS:**

This is a prospective experimental study consisting of two independent, non-randomized groups: fibromyalgia (n=10) and healthy control (n=10). The research was conducted in person at the Unidade Saúde Escola of the Federal University of São Carlos, Brazil, during the period of June 2023–June 2024. The conditioned pain modulation test was administered to all patients in both groups. Twice a week (for 3 months), patients with fibromyalgia performed three sets of each exercise, with 10 repetitions, 40 s of muscle tension, and a 60-s interval between the sets. Subsequently, we compared the conditioned pain modulation test using the Student's t-test.

**RESULTS:**

Significant between-groups differences (p≤0.05) and large effect sizes (d≥0.8) that were observed at baseline (at times 2, 3, and 4) were not observed after 3 months of strength training, indicating that the intervention was able to improve the conditioned pain modulation in patients with fibromyalgia. Although the within-group comparisons (pre- and posttreatment) showed an absolute difference of 6.35 in central sensitization, this was not statistically significant (p≥0.05) and had a small effect size (d=0.27).

**CONCLUSIONS:**

The conditioned pain modulation of fibromyalgia patients appears to become similar to that of healthy individuals after 3 months of strength training.

## INTRODUCTION

Fibromyalgia is a condition characterized by a range of symptoms including widespread chronic pain, fatigue, sleep disturbance, cognitive dysfunction, depression, and others, with increased responses to nociceptive and somatic stimuli^1^. Fibromyalgia affects approximately 5% of the world's population, with a higher incidence in women, and generally presents between the ages of 30 and 35 years; however, it is still difficult to diagnose due to the complexity of the symptoms^2^.

Thus, the lack of a clear definitive diagnosis for ­fibromyalgia delays adherence to treatment, but interventions focus on symptom improvement to promote quality of life in these patients^2^. Treatments include pharmacologic therapy (medications with neurologic, analgesic, and muscle effects) and/or non-pharmacologic therapy (pain education, cognitive-behavioral therapies, and exercise)^2^.

Recommendations for exercise in the management of fibromyalgia point to the safety of strength training^3^. Although all types of exercise have been shown to be beneficial, strength training is of particular interest because it is a type of exercise that addresses patient preference and thus evidence-based practice^3^. However, there are still no studies that clearly describe the effects of strength training on pain modulation mechanisms in patients with fibromyalgia^4^.

An important phenomenon in understanding the ­mechanisms of pain in fibromyalgia is conditioned pain modulation (CPM), the function of which reflects changes in central descending inhibitory systems^4^. CPM is a phenomenon in which one pain (pain x) reduces the perception of another pain (pain y)^4^. This occurs because nociceptive neurons in the spinal and dorsal horns of the trigeminal nerve are inhibited by noxious stimulation far from the excitatory receptive field of the neurons. This phenomenon is assessed by the CPM test^5^.

The CPM test is an objective measure of the body's pain inhibitory pathway, known as the "pain inhibits pain" phenomenon^6^. Studies using this test in patients with chronic pain and comparing them to healthy individuals have reported that fibromyalgia patients have a lower CPM effect^7^. Based on these findings, Potvin et al.^7^ suggested that the perception of pain as observed by CPM needs to be scientifically investigated^7^.

In light of this, we note that there are still no studies investigating this outcome in patients with fibromyalgia undergoing strength training, highlighting a gap in this context. Therefore, we aimed to evaluate the CPM of patients with fibromyalgia and compare it to that of healthy individuals before and after 3 months of strength training. The hypothesis was that the CPM of patients with fibromyalgia after 24 sessions of strength training (3 months) would be similar to that of healthy individuals.

## METHODS

### Design and ethical aspects

This prospective experimental study was conducted on two independent, non-randomized groups: the fibromyalgia group and the healthy control group (which did not receive any intervention). For this reason, there was no need for randomization and/or allocation concealment, as fibromyalgia patients were exclusively in the experimental group (only one type of intervention), while healthy patients were in the control group.

The research was conducted in person at the Unidade Saúde Escola (USE) of the Federal University of São Carlos (UFSCar), Brazil, during the period of June 2023–June 2024. All procedures were previously approved by the Research Ethics Committee (report number: 5499078). All respondents participated in this study freely and with consent. All experiments were performed in accordance with relevant guidelines and regulations.

The research was publicized through social media (WhatsApp™, Facebook™, Instagram™, and Twitter™), through the university's dissemination channels, and through flyers and posters in public health services in the city of São Carlos (SP), Brazil. After voluntary and informed completion of the Google Forms™, each person with fibromyalgia was individually invited to participate in the in-person assessments, which included CPM and sample characterization.

### Sampling and study size

The power calculation was performed a priori with the G*Power software (version 3.1.9.7)™ using the t-test for comparisons between two independent samples (fibromyalgia vs. healthy). We used Cohen's d=1.2 (in between-group comparisons for the outcome of CPM), alpha=0.5, power=0.80, allocation ratio of 1/1, non-centrality of the parameter δ=2.6832816, and critical t=1.7340636^8^. Thus, the sample was estimated at 20 patients divided into two groups (fibromyalgia, n=10; healthy, n=10).

### Group eligibility

In the fibromyalgia group, we included patients with a diagnosis of fibromyalgia according to the fibromyalgia diagnostic criteria of 2016^9^, who were aged between 18 and 55 years, who were residents of the city of São Carlos (SP) (Brazil), and who were not involved in other exercise programs.

In the healthy group, we included patients without a diagnosis of fibromyalgia, without any other pain (acute or chronic), aged between 18 and 55 years, living in the city of São Carlos (SP) (Brazil), and who self-reported no physical activity.

We did not include patients with neurological conditions that would interfere with the assessments, such as paralysis, significant sensory changes, and changes in the level of consciousness/understanding; advanced joint disease; suspected thrombosis, heart disease, and immediate postoperative period; pregnancy; alcohol and illicit substance abuse; and active cancer. This information was screened from self-reported responses on Google Forms™.

### Conditioned pain modulation

The CPM test was administered to all patients in both groups^4^. This assessment verifies the perception of pain by a conditioned stimulus^10-12^. We assessed pain intensity in patients with fibromyalgia using the Numeric Pain Rating Scale, an instrument validated by Ferreira-Valente et al.^13^. This scale consists of a series of numbers (from 0 to 10), with 0 representing no pain and 10 representing the worst pain.

To identify the phenomenon of pain inhibition, we used the following equation: ([post-test score/pre-test score]×100) . Values >100 indicate an increase in the pressure pain threshold, representing pain inhibition by modulation of another pain (ischemic stimulus). Scores<100 indicate possible central sensitization^14^.

### Maximum dynamic strength

We identified the maximum dynamic strength using the one-repetition maximum (1-RM) test. The results of this test, in addition to being reliable (intraclass correlation coefficient [ICC]=0.90)^15^, are widely used to control the intensity of exercise based on the percentage of maximum strength in patients with fibromyalgia^15^. The patients performed 10 repetitions of the movement without load for musculoskeletal warm-up purposes and to understand the technique; after 1 min of rest, they performed 3–5 self-reported maximum repetitions, and then after a 3-min rest, they performed the 1-RM test^16^.

In addition, as an alternative to corroborate the test results, we estimated the maximum dynamic strength for the 1-RM using the mathematical equation proposed by Brzycki: 1-RM=submaximal load in kg/(1.0278–0.0278×number of repetitions) ^17^.

### Intervention (strength training)

Before the first strength training session, there was a period of familiarization, followed by a 1-RM in each of the suggested strength training exercises. All sessions took place individually, in a private room with lighting and air conditioning at 23°C. All exercises were led by a trainer who had a bachelor's degree in physical education with experience in exercise for patients with chronic pain (Pontes-Silva A).

The patients trained nine muscle groups (gluteus maximus, quadriceps, hamstrings, biceps brachii, triceps brachii, pectoralis major, triceps surae, deltoid, and latissimus dorsi) through six different exercises in the following order: I—knee extension machine, II—seated calf raise, III—leg press, IV—incline bench press, V—seated row machine, and VI—lateral dumbbell raise^18^.

The intensity of the exercises (total load in kilograms), the interval between sets (rest in seconds), the volume (number of sets, repetitions, and time under tension), and the frequency of strength training were controlled by the researcher with experience in exercise for patients with chronic pain. Twice a week (for 3 months), patients with fibromyalgia performed three sets of each exercise, with 10 repetitions, 40 s of muscle tension, inspiration during the eccentric phase of the movement, and a 60-s interval between the sets^19^.

### Load progression

The initial parameter for the initial intensity (moderate) in the strength training was considered 50% of the maximum strength, through the 1-RM test in the proposed exercises. We identified the load in kilograms and evaluated it every 4 weeks. The muscular strength of the patients and the intensity of each exercise received an increased adjustment of 20% of the value observed in kilograms by the 1-RM test (i.e., in the first month 50%; in the second month 70%; and in the third month 90% of the maximum strength)^19,20,^.

We added a 20-s rest interval between the sets after the intensity progression in order to maintain the same volume of exercises in the strength training (in the first month, it was 60 s, in the second month, it was 80 s, and in the third month, it was 100 s), considering that with the same total work volume (sets, repetitions, and time under tension), higher intensities require longer rest intervals^19^.

### Statistical analysis

We compared quantitative variables using the intention-to-treat principle^21^ and the Student's t-test (with alpha ≤0.05) for paired and independent samples with normal distribution analyzed using histograms in the Shapiro-Wilk test. Thus, comparisons were described as mean, standard deviation, mean difference (i.e., delta [Δ]), and confidence interval (CI) of the difference (95%CI). SPSS™ software (version 17, Chicago, Illinois, USA) was used for data processing. In addition, Cohen's d was used to assess effect sizes when comparing two means: 0.2=small effect, 0.5=moderate effect, and 0.8=large effect^8^.

## RESULTS

The total sample consisted of 10 women with fibromyalgia and 10 healthy women. Notably, 70% of the women with fibromyalgia were taking medication and had a Widespread Pain Index average of 10.50 (±2.59) distributed among the right and left sides and posterior axial region, with a symptom severity average of 6.50 (±1.26) distributed among fatigue, awakening malaise, and cognitive symptoms. We observed a statistically significant difference (p≤0.05) and a large effect size (d≥0.8) in age, body mass, body mass index (BMI), and stature when comparing the groups (Table 1). This clinical difference between the samples allowed comparison of the main outcome of this study (i.e., central sensitization).

**Table 1:** Sample characterization.

Variables	Fibromyalgia	Healthy	Δ	d	95%CI	
Age (years)	45.00 (8.80)	23.90 (2.13)	21.10*	3.29#	15.08	27.11
BMI (kg/m^2^)	30.31 (5.33)	23.60 (3.48)	6.71*	1.49#	2.48	10.94
Body mass (kg)	80.78 (15.63)	62.90 (8.26)	17.88*	1.43#	6.13	29.62
Stature (m)	1.63 (0.03)	1.63 (0.05)	0.00	0	-0.04	0.03
WPI (score)	10.50 (2.59)	n/a	n/a	n/a	n/a	n/a
SSS (score)	6.50 (1.26)	n/a	n/a	n/a	n/a	n/a

BMI: body mass index; CI: confidence interval; SSS: Symptom Severity Scale. WPI: Widespread Pain Index;

Δ delta;

d Cohen's d;

N/a not applicable.

* Significant difference: independent-samples t-test (p≤0.05).

# Large effect size: Cohen's d≥0.8. Mean (standard deviation).

Central sensitization showed an insignificant difference (p≥0.05) and a small effect size (d=0.26) between groups at both baseline and after treatment; however, the significant differences between groups observed at baseline (at times 2, 3, and 4) disappeared after 3 months of strength training, ­making the groups statistically similar (Table 2).

**Table 2:** Comparisons between groups.

CPM		Fibromyalgia	Healthy	Δ	d	95%CI	
Pre exercise (baseline)							
	Time 1 (kg)	4.28 (2.48)	5.37 (1.57)	1.09	0.52	-3.04	0.86
	Time 2 (kg)	4.12 (1.50)	6.50 (1.97)	2.38*	1.35#	-4.02	-0.73
	Time 3 (kg)	3.96 (1.75)	5.63 (1.20)	1.67*	1.11#	-3.08	-0.25
	Time 4 (kg)	3.94 (1.98)	5.66 (1.63)	1.72*	0.94#	-3.42	-0.01
Central sensitization (<100)		100.47 (28.60)	108.02 (28.44)	7.55	0.26	-34.35	19.25
Post exercise							
	Time 1 (kg)	4.59 (1.88)	5.37 (1.57)	0.77	0.45	-2.41	0.85
	Time 2 (kg)	5.25 (2.49)	6.50 (1.97)	1.24	0.55	-3.35	0.86
	Time 3 (kg)	4.95 (2.20)	5.63 (1.20)	0.67	0.38	-2.34	0.99
	Time 4 (kg)	4.91 (2.29)	5.66 (1.63)	0.74	0.37	-2.61	1.12
Central sensitization (<100)		106.82 (15.84)	108.02 (28.44)	1.20	0.05	-22.83	20.43

CI: confidence interval; CPM: conditioned pain modulation;

Δ delta;

d Cohen's d.

* Significant differences: independent-samples t-test (p≤0.05).

# Large effect size: Cohen's d≥0.8. Mean (standard deviation).

Although the within-group comparisons (pre- and posttreatment) showed an absolute difference of 6.35 in central sensitization, this was not statistically significant (p≥0.05) and had a small effect size (d=0.27) (Table 3).

**Table 3:** Comparisons within groups (pre- and postexercise).

CPM	Pre exercise	Post exercise	Δ	d	95%CI	
Time 1 (kg)	4.28 (2.48)	4.59 (1.88)	-0.31	0.14	-2.63	2.00
Time 2 (kg)	4.12 (1.50)	5.25 (2.49)	-1.13	0.55	-3.01	0.74
Time 3 (kg)	3.96 (1.75)	4.95 (2.20)	-0.99	0.49	-2.44	0.45
Time 4 (kg)	3.94 (1.98)	4.91 (2.29)	-0.97	0.45	-2.44	0.49
Central sensitization (<100)	100.47 (28.60)	106.82 (15.84)	-6.35	0.27	-33.22	20.52

CI: confidence interval. CPM: conditioned pain modulation;

Δ delta;

d Cohen's d.

Comparisons show no significant differences: paired samples t-test (p≥0.05). Large effect size: Cohen's d≥0.8. Mean (standard deviation).

## DISCUSSION

Our hypothesis was that the CPM of patients with ­fibromyalgia after 24 sessions of strength training (3 months) would be ­similar to that of healthy individuals. Although the central sensitization index showed an insignificant difference and a small effect size between groups at baseline and after treatment, the significant differences between groups observed at baseline (at times 2, 3, and 4) disappeared after 3 months of strength ­training—indicating that the intervention was able to improve the observed outcome (CPM).

We know that CPM is a centrally processed measure of the net effect of the descending pain pathway^4,5^. We also know that strength training promotes biological adaptations in the central and peripheral nervous systems^22,23,24^, but these adaptations are poorly understood in fibromyalgia studies. Our study suggests that 3 months of strength training with 2 weekly sessions is capable of producing adaptations resulting from neuroplasticity.

Because this is the first study to evaluate CPM in fibromyalgia patients and compare it to that of healthy individuals before and after 3 months of strength training, the discussion is reduced, but our results open the possibility for studies of the nervous system applied to fibromyalgia regarding the response time to neurological adaptations resulting from strength training.

Furthermore, it is also necessary to discover other amounts of time (e.g., 1, 2, 4, or 5 months), other types of exercise (e.g., walking and water aerobics), as well as other proposals for ­physical effort in strength training since this study only investigated strength training with progressive linear periodization^25^.

This study has limitations that need to be addressed. Our sample consisted only of women with fibromyalgia, and we are not sure whether these results can be generalized to men. Therefore, we recommend further studies in this regard.

## CONCLUSION

The CPM of fibromyalgia patients appears to become similar to that of healthy individuals after 3 months of strength training. Further studies should compare statistically different samples and test whether the difference will disappear after treatment.

## Data Availability

The datasets generated and/or analyzed during the current study are available from the corresponding authors upon reasonable request.
